# A choice, not an obligation: Releasing scientific software as open source should be the responsibility of the authors

**DOI:** 10.1038/s44319-023-00039-9

**Published:** 2024-01-02

**Authors:** I Kappas, VJ Promponas, CA Ouzounis

**Affiliations:** 1 School of Biology, Aristotle University of Thessalonica, Thessalonica, Greece; 2 Department of Biological Sciences, University of Cyprus, Nicosia, Cyprus; 3 School of Informatics, Aristotle University of Thessalonica, Thessalonica, Greece

**Keywords:** Computational Biology, Science Policy & Publishing

## Abstract

The timing of making software open source should not be dictated by journal guidelines.

**Figure:**
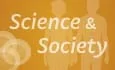

Scientists who develop software as part of their work often have to make a decision about whether and when to release their code as open source. Many of us, in alignment with the current emphasis on transparency and reproducibility, actively endorse and engage in the open-source movement, not only for code but also for data, referred to as open source and open data, respectively, albeit two quite different things. Overall, the movements promoting open source and data, collectively known as open science, have gained significant momentum in recent years across many scientific disciplines (Fortunato and Galassi 2021).

Many of us, in alignment with the current emphasis on transparency and reproducibility, actively endorse and engage in the open-source movement…

However, there are cases where the precise timing of releasing code as open source is crucial, particularly concerning its publication in peer-reviewed journals. This dilemma prompts the question of who decides when software should be made open source (Melvin et al, 2022). Historically, and perhaps in an arbitrary manner, this responsibility has been taken over by publishers, editors, and scientific journals.

## Open-source software

Releasing software as open source confers a range of discernible advantages. First, it enables the community to dissect and rigorously examine the software, thereby advancing the twin principles of transparency and dependability. Second, it provides the developers with precious insights from user-reported issues, which expedites the resolution of problems. Not surprisingly, open-source software commonly witnesses a surge in popularity and accessibility, rendering it available to a broader and diverse audience.

Moreover, open-source software allows for extensive customization by users to make adjustments to parameters, expand memory allocation, or incorporate more complex processes than initially envisioned (Silva et al, 2017). The degree of community involvement is regulated by developers or defined by open-source licenses, ensuring that the software can evolve and be extended without compromising its core code. It’s worth emphasizing that maintaining open-source projects requires substantial human and computational resources, which are mostly supported through research and development grants from public sources (Zielinski et al, 2019).

## Release time

The opportune moment for releasing code is typically the acceptance of the manuscript for publication in a journal after rigorous evaluation by reviewers and editors. Remarkably, in the context of software articles, the manuscript assessment process has quietly evolved into an implicit benchmark for quality assurance. Thus, some publishers have adopted guidelines that require disclosure of the software as open source during the initial paper submission (Table 1), a paradigm akin to but distinct from open-data policies (Peccoud, 2021). This shift, in essence, transmutes the role of reviewers, traditionally focused on evaluating the structure, content, and interpretation of the submitted work, into that of software testers (Gardner et al, 2022).

**Table 1:** Scientific journals and/or publishers involved in the publication of studies with research software.

Required at acceptance	Recommended	Required at submission
BMC Bioinformatics	PLoS Comp Biol	Bioinformatics
EMBO Press	PLoS ONE	
Proc Natl Acad Sci USA	Nature	
Science		
Nature		

Those policies are classified into three categories with regard to open source.

This change presupposes that authors have to release their code as open source, often required by the ‘Instructions for Authors’ through statements such as “… software and code must be open source”. But the imposition of mandatory release policies during the submission process poses two challenges to reviewers. First, it compels them to inspect and test the software, which, at least in principle, extends their traditional responsibilities beyond evaluating the soundness and quality of the results and their interpretation. Second, reviewers are asked to undertake software testing without any compensation, despite it being a time-consuming activity that is usually well-rewarded in the industry. In the case of scientific software, this responsibility should primarily rest with the authors and their collaborators; in fact, a mandatory release during or before submission shifts the burden of quality assurance from software developers to reviewers, potential users and consumers (Resnik and Elliott, 2016). In the end, scientists shoulder the primary responsibility for the code’s characteristics, precision, and availability to the wider scientific community.

In the end, scientists shoulder the primary responsibility for the code’s characteristics, precision and availability to the wider scientific community.

## Enforcing code release

Let us contemplate a common scenario: a paper that exclusively revolves around software and pipelines, which serves as a *bona fide* announcement. Depositing it as a preprint or releasing it upon submission can remove this distinct quality. Code release is a critical moment for the uptake of software by a community and a common strategy in the software industry that the authors are barred from in this case. Publishing a software article as a preprint or releasing the code as open source upon submission omits this valuable element of “surprise”, which might have a significant academic impact.

In cases where the software development project has already garnered recognition or reissuance, its announcement as a preprint actually nullifies any potential advantages offered by a full publication in a peer-reviewed scientific journal article (Cabanac et al, 2021). In these situations, the significance of reviewers is diminished, and the assurance of publication and originality is no longer upheld. For example, if a reviewer at the outset commits to evaluating the work but subsequently disengages from the process for any reason, or if the work is not accepted, its content remains accessible to the reviewer. Moreover, if the code has been forced to be released upon submission, it becomes “open”, even if to a limited audience, and may potentially be disseminated as a URL on social networks or through intermediaries to a wider audience.

This scenario exposes not only the author but also their team, institution, collaborators, as well as publishers, editors or reviewers to risks, as the material has been leaked without formal publication and peer review. Such a condition goes against the principles and ethics of the scientific community (Hamilton et al, 2020).

In contrast, when reviewers concentrate their assessment solely on the structure and content of a publication while pointing out any deficiencies to the editors, the journal effectively shifts the responsibility for process integrity to the authors. This approach guarantees developers the authority to make key decisions about releasing their software as open source upon acceptance, should they believe it serves the best interests of the scientific community (Chambers, 2020).

In taking this approach, authors not only gain favor within the scientific community but also retain control over the timing of their announcement. Programmers and ‘beta-testers’, often funded by public sources, can be confident that their intellectual property will not be inappropriately disclosed or misused, regardless of the open-source license governing it (Pearce, 2020). Various solutions for facilitating open science in this context have been proposed (Cohen-Sasson and Tur-Sinai, 2022). Hence, at the pivotal stage of the submission-to-acceptance-to-publication process, authors should be the ones to choose when to make their software publicly accessible.

… at the pivotal stage of the submission-to-acceptance-to-publication process, authors should be the ones to choose when to make their software publicly accessible.

Developers may and should willingly shoulder the complete responsibility for maintaining and expanding the open-source framework, driven by choice rather than obligation. This not only garners them appreciation and respect from the broader community but also positions them favorably for potential funding from public resources and equitable consideration in the grant review process. Funding agencies may even envisage a reward scheme for publishing open versus non-open source as a criterion and not as a strict requirement. Publishers can also require open access, but this should be upon acceptance.

## Control by developers

In summary, the decision of whether or when to transition scientific software into open source, influenced by factors related to development and maintenance, firmly rests with its developers (Noor, 2022). In some cases, where public funds are lacking or difficult to obtain, commercialization can be used as an alternative funding source to further support development. Publishers may lack a detailed understanding of the specific circumstances of this important option (Koru et al, 2007). While many of us strongly advocate for open-source principles, there are cases where their immediate adoption may not be viable due to resource limitations and other factors (Yapa and Bärnighausen, 2018). Releasing the code as open source at acceptance may be the optimal strategy for software papers. This proposal is not too dissimilar from the ‘embargo’ policy that DNA sequence databases have in place to secure the timing and control of access by the original contributors, a mechanism that is not currently available in software repositories widely used for open-source code deployment.

While many of us strongly advocate for open-source principles, there are cases where their immediate adoption may not be viable due to resource limitations and other factors.

Regardless, the timing for transitioning code to open source should remain under the control of scientists and their collaborators and should not be an absolute prerequisite for publication. The success and effectiveness of software development projects frequently depend on making the appropriate publishing decisions at the right moment rather than rigidly adhering to prescribed processes that might carry a flair of political correctness within scientific journals and publishers (Horbach and Halffman, 2020). After all, good citizenship does not automatically guarantee quality. The sustainability of open source is at the hands of the developers when they decide to communicate their work in a scientific paper upon acceptance, and beyond.

## Supplementary information

Peer Review File
