# Supplementary material for: A choice, not an obligation: Releasing scientific software as open source should be the responsibility of the authors
Source: EMBO Rep. 2024 Jan 2;25(2):464–6. doi: 10.1038/s44319-023-00039-9 (PMC10897218; doi:10.1038/s44319-023-00039-9)
Supplement: Supplementary file 1 — Peer Review File [file 44319_2023_39_MOESM1_ESM.pdf]

EMBO reports

A choice, not an obligation

Ilias Kappas, Vasilis Promponas, and Christos Ouzounis
DOI: 10.15252/embr.202358541

Corresponding author(s): Christos Ouzounis (cao@csd.auth.gr)

**Review Timeline:**	Submission Date:	23rd Nov 23
	Accepted:	13th Dec 23

Editor: Holger Breithaupt

Transaction Report: This article was editorially reviewed.
